# Who benefits? Uncovering hidden heterogeneity of treatment effects in adaptive trials using Bayesian methods: a systematic review

**DOI:** 10.1186/s13063-025-09291-x

**Published:** 2025-11-25

**Authors:** Rachel Giblon, Chengyang Gao, Kuan Liu, Yongdong Ouyang, Jessie Cunningham, Allen L. Pimienta, Ewan C. Goligher, Anna Heath

**Affiliations:** 1 Institute of Health Policy, Management, and Evaluation, University of Toronto Dalla Lana School of Public Health, Toronto, ON Canada; 2 Division of Biostatistics, University of Toronto Dalla Lana School of Public Health, Toronto, ON Canada; 3 Child Health Evaluative Sciences, The Hospital for Sick Children, 686 Bay Street, Toronto, ON Canada; 4 Department of Statistical Science, University College London, London, UK; 5 SickKids Hospital Library, The Hospital for Sick Children, Toronto, ON Canada; 6 Department of Family and Community Medicine, University of Toronto, Toronto, ON Canada; 7 Interdepartmental Division of Critical Care Medicine, University of Toronto, Toronto, ON Canada; 8 Division of Respirology, Department of Medicine, University Health Network, Toronto, ON Canada

**Keywords:** Bayesian statistics, Heterogeneity of treatment effect (HTE), Adaptive clinical trials, Statistical methods, Subgroup analysis, Personalized medicine, Health equity, Trial design

## Abstract

**Background:**

Adaptive clinical trials increasingly aim to detect heterogeneity of treatment effect (HTE) to guide personalized care. However, most adaptive designs rely on predefined subgroups and are limited in their ability to uncover unknown or complex sources of HTE. Bayesian statistical methods offer a flexible alternative, enabling real-time learning and adaptation within trials. This review evaluates Bayesian methods used to detect hidden HTE in adaptive clinical trials, with attention to their methodological innovations, operating characteristics, and consideration of equity and inclusion in trial design.

**Methods:**

We conducted a systematic search of MEDLINE, Embase, and other databases to identify original studies that developed Bayesian methods for detecting unknown HTE within adaptive clinical trial designs. Eligible studies were reviewed and synthesized based on design features, statistical methodology, operating characteristics, reproducibility, and whether equity-related factors were explicitly considered. Equity considerations included whether studies incorporated variables related to underrepresented populations—such as age, sex, race/ethnicity, or geography—examined intersectional subgroup effects, or explicitly framed their methods as tools to address health disparities.

**Results:**

Of 2826 screened records, seven studies met inclusion criteria. Bayesian methods included random partition models, spatial models, logistic regression with dimension reduction, adaptive randomization using machine learning classifiers, and adaptive enrichment or platform designs incorporating model averaging or latent subgroup estimation. In simulation studies, these methods often showed improvements in subgroup detection, efficiency, or power relative to non-Bayesian comparators. None were tested using real-world trial data. Reproducibility was limited overall, with analytic code only available for the three most recent studies. Notably, none explicitly framed their methods as tools to address inequities in treatment outcomes across population subgroups.

**Conclusions:**

The small number of simulation-based studies illustrates preliminary but promising directions for applying Bayesian methods to detect HTE in adaptive clinical trials. While these approaches demonstrate potential to enhance trial adaptability, scalability, and inclusiveness, current evidence remains limited and largely conceptual. Incorporating an equity lens into future methodological development, alongside greater emphasis on empirical validation and open science practices, will be essential to determine their practical value in advancing equitable clinical research.

**Supplementary Information:**

The online version contains supplementary material available at 10.1186/s13063-025-09291-x.

## Background

Clinical trials are the cornerstone of evidence-based medicine, guiding decisions about which interventions are safe and effective for patients [1]. However, many trials are designed to estimate an average treatment effect across a broad population, assuming that this average applies uniformly to all participants. In reality, treatment effects often vary across individuals due to biological, clinical, and social differences—a phenomenon known as heterogeneity of treatment effect (HTE). While the population average treatment effect may be appropriate when evaluating a therapy’s overall efficacy, understanding variation in response becomes critical when treatment decisions are intended to be tailored to individual patients or subgroups. When HTE is present, applying average results to all patients can lead to ineffective or even harmful care, particularly for those who differ meaningfully from the “average” trial participant. Growing awareness of this issue has driven efforts to better account for HTE in clinical research, with the goal of supporting more personalized and effective care [2, 3].

Importantly, addressing HTE is not only about improving precision—it also has potential implications for equity. When subgroup differences in treatment response go unrecognized or unreported, trials may inadvertently reinforce health inequities, particularly for groups historically underrepresented or underserved in research, such as racial and ethnic minorities, older adults, and people with disabilities [4, 5]. These groups are often considered a priori in equity-focused frameworks like NIHR-INCLUDE, which defines underserved populations as those whose inclusion in research is disproportionately low relative to their healthcare burden or for whom treatment effects remain uncertain due to a lack of subgroup-specific data [6]. However, many equity-relevant differences in treatment response may not align neatly with predefined categories like sex, race, or disability status. In such cases, relying solely on stratified subgroup analyses can obscure clinically meaningful heterogeneity. From this perspective, failure to account for HTE—whether across known or previously unrecognized subgroups—may contribute to knowledge gaps and limit the generalizability of findings, highlighting a potential disconnect between trial populations and real-world patients [7, 8]. Greater attention to HTE is therefore important for improving the relevance and applicability of trial findings and for supporting more inclusive research practices.

HTE can be evaluated in two main ways: (1) by analyzing treatment effects across *predefined* subgroups (e.g., based on biomarker status or disease stage), or (2) by identifying *previously unknown* subgroups with differential treatment responses. The first approach is common in fields like oncology, where biomarkers often guide therapy, and has been widely studied in the context of adaptive enrichment and predefined subgroup analyses [9], with Bayesian methods increasingly used to optimize treatment decisions within known subgroups [10–13]. However, in fields such as critical care and emergency medicine where predictive biomarkers are often lacking, the second approach—which dynamically identifies hidden subgroups during the trial—is especially valuable. These settings also disproportionately serve individuals facing barriers to preventive care, making it even more urgent to detect overlooked sources of treatment variation. By revealing response patterns tied to underserved populations—even when these groups are not explicitly defined—subgroup discovery can play a crucial role in advancing equity.

Traditionally, hidden subgroups have been identified through a two-step process: discovery in observational data, followed by validation in randomized trials. For example, Calfee et al.’s latent class analysis of patients with acute respiratory distress syndrome identified two distinct biological subphenotypes with different treatment responses [14]. However, it took nearly a decade before these findings were prospectively evaluated in the PANTHER trial [15]. Such delays limit the clinical impact of subgroup discovery, with underserved populations often waiting the longest for evidence to guide care [4–6]. Adaptive clinical trials offer a promising alternative to this traditional two-step model by enabling in-trial detection of HTE and responsive trial modifications [16]. Regulatory bodies such as the U.S Food and Drug Administration (FDA) increasingly supported such innovation [5], encouraging adaptive designs that allow for mid-trial eligibility expansions and subgroup analyses to better reflect the populations who will ultimately use the treatment. These strategies align with the INCLUDE framework’s call for proactive identification of barriers to inclusion and support more flexible, responsive, and inclusive trial designs [6].

However, many current adaptive trials rely on frequentist methods, such as stratified analyses or data-driven subgroup detection procedures [17]. These approaches face well-known limitations when used to detect hidden HTE during an ongoing trial, including inflated type I error due to multiple comparisons [18], high data demands, and limited interpretability of subgroup findings [19, 20]. Moreover, frequentist methods are not well-suited to incorporating prior knowledge or adapting to evolving subgroup structures as new data accumulate [9, 21–24]. While a wide range of approaches exist to study HTE—including methods from the broader frequentist literature [17, 25]—Bayesian methods offer a flexible probabilistic framework that supports continuous updating of treatment effect estimates and subgroup definitions over the course of a trial [26], even in hybrid designs that combine frequentist-style subgroup detection with Bayesian modeling [27]. This flexibility is particularly valuable in the context of equity: Bayesian models can yield more stable estimates in small or underserved subpopulations, incorporate external evidence about disparities, and detect unanticipated sources of treatment heterogeneity that may otherwise go unnoticed [10, 12]. By supporting real-time learning and adaptation, Bayesian methods enhance the potential of adaptive trials to both increase efficiency and better reflect the diversity of the patient populations they aim to serve.

To our knowledge, this is the first systematic review to evaluate Bayesian methods for detecting hidden HTE within adaptive trial designs. By synthesizing current methodological approaches and performance outcomes, we aim to provide actionable insights for trialists seeking to implement Bayesian HTE detection in real-world adaptive trials. We further explore how these methods may support more equitable trial practices by improving identification of differential treatment responses across diverse and underserved subpopulations.

## Methods

### Key concepts in adaptive trials

Adaptive clinical trials enable pre-specified modifications to study protocols—such as sample size re-estimation, treatment allocation adjustments, or changes in patient eligibility—based on accumulating interim data [16]. These designs enhance trial efficiency, ethical decision-making and responsiveness to emerging evidence. Unlike traditional fixed-protocol trials, adaptive trials incorporate planned flexibility and require rigorous pre-specification of adaptation rules.

One key strategy is *adaptive randomization*, where treatment allocation probabilities change during the trial based on accruing data [16, 28, 29]. Common approaches include:

(i) Restricted randomization: Balances treatment groups based on prior assignments.
(ii) Covariate-adaptive randomization: Adjusts allocation based on both prior assignments and patient characteristics.
(iii) Response-adaptive randomization: Modifies randomization probabilities based on observed interim responses.
(iv) Covariate-adjusted response adaptive (CARA) randomization: Combines covariate-adaptive and response-adaptive strategies for dynamic adjustments.

Another important approach is *adaptive enrichment*, which modifies eligibility criteria mid-trial to focus on subgroups showing greater potential benefit—often identified based on genetic, biomarker, or clinical features [16, 30, 31]. Adaptive Signature Designs (ASD) represent a specialized method where exploratory analyses identify treatment-responsive subgroups, which are then validated in a subsequent confirmatory phase [16, 32]. ***Platform trials*** evaluate multiple therapies within a single disease framework using a shared control arm, allowing treatments to enter or leave the trial adaptively as evidence accumulates [16, 33]. *Basket trials*, by contrast, assess a single therapy across multiple disease types or molecular subgroups [33], enabling evaluation of treatment effects in biologically defined populations rather than traditional disease categories. Although most adaptive designs apply stopping rules for superiority or futility within pre-specified strata [9, 22], emerging Bayesian methods extend this framework by allowing subgroup definitions to evolve dynamically from the data.

### Bayesian vs. frequentist approaches to HTE detection

Bayesian methods treat unknown parameters, such as treatment effects, as random variables with associated probability distributions. They allow prior knowledge, from expert opinion or previous studies, to be formally incorporated and continuously updated with accruing trial data, producing posterior distributions that reflect both sources of information [34, 35]. This framework facilitates real-time trial adaptations, including adjustments to treatment allocation, stopping boundaries, or sample size, based on evolving evidence [16, 36]. Bayesian priors also increase the effective sample size, which is valuable when assessing treatment effects in small subgroups prone to low power. Additionally, Bayesian models naturally accommodate hierarchical structures, allowing information-sharing across subgroups while modeling subgroup-specific treatment effects [10, 12]. Outputs such as credible intervals offer intuitive probabilistic interpretation [37], supporting clinical-decision making and personalized trial adaptations [26]. These benefits are balanced by practical limitations, including bias or reduced effective sample size from poorly specified priors, the computational intensity of high-dimensional adaptive models, and the risk of overinterpreting posterior probabilities when data are sparse.

In contrast, frequentist approaches treat unknown parameters as fixed quantities and rely solely on observed data for inference. They generate confidence intervals and hypothesis tests based on long-run frequency properties without integrating prior knowledge [34, 35]. While frequentist methods remain widely used and are familiar to regulators, they are less suited for complex adaptive designs or real-time HTE detection, especially when modeling evolving subgroup structures or integrating prior evidence [34, 35, 37]. This review focuses specifically on Bayesian methods for detecting hidden HTE in adaptive trials contexts, given their growing methodological and practical advantages.

### Search strategy

A systematic literature search was conducted in two stages. The initial search was performed on January 18, 2024, using Ovid MEDLINE(R) and Epub Ahead of Print, In-Process, In-Data-Review & Other Non-Indexed Citations and Daily < 1946 to January 17, 2024 >; and Ovid Embase + Embase Classic (1974–2024 week 02). The search was updated on October 7, 2025, using the same databases to capture newly published studies. Both searches combined controlled vocabulary, such as the National Library of Medicine’s MeSH (Medical Subject Headings) and Emtree Subject Headings (Embase), and relevant keywords related to Bayesian methods, HTE, and adaptive clinical trials, without restrictions on publication date. Search results from all databases were imported into Covidence [38] for deduplication and screening. Details of the full search strategy are available in Additional Table 1. Key terms included “Bayesian statistical methods”, “adaptive clinical trials”, “heterogeneity of treatment effect”, and “subgroup analysis”.

### Study selection and quality assessment

Study selection followed a two-step process. For both the initial (RG, CG) and updated (RG, AH) searches, two independent reviewers screened titles and abstracts against predefined inclusion and exclusion criteria (Table 1). Full-text articles marked as “include” or “unsure” were retrieved for further eligibility assessment. Agreement between reviewers was substantial (Cohen’s *κ* = 0.70). Discrepancies were resolved through discussion, with a third reviewer consulted as needed for final decisions. We restricted inclusion to studies developing Bayesian methods for HTE detection in adaptive trials, excluding frequentist and prespecified subgroup approaches to ensure a focused synthesis of recent Bayesian innovations.

**Table 1:** Inclusion and exclusion criteria for systematic literature search

Inclusion criteria	Exclusion criteria
• Original research articles	• Literature reviews, published commentaries and letters
• English publications	• Language other than English
• Primary analysis focuses on the development of a novel HTE detection statistical design for clinical trials	• Primary analysis reports the results of a clinical trial or observational study
• Bayesian methodology used to identify previously unknown HTE subgroups	• Frequentist methodology used to identify HTE subgroups
• Methods that aim to improve subgroup selection/responder analysis	• Analyses of HTE only among pre-specified subgroups
	• N-of-1 studies

### Reporting standards

**Fig. 1:**
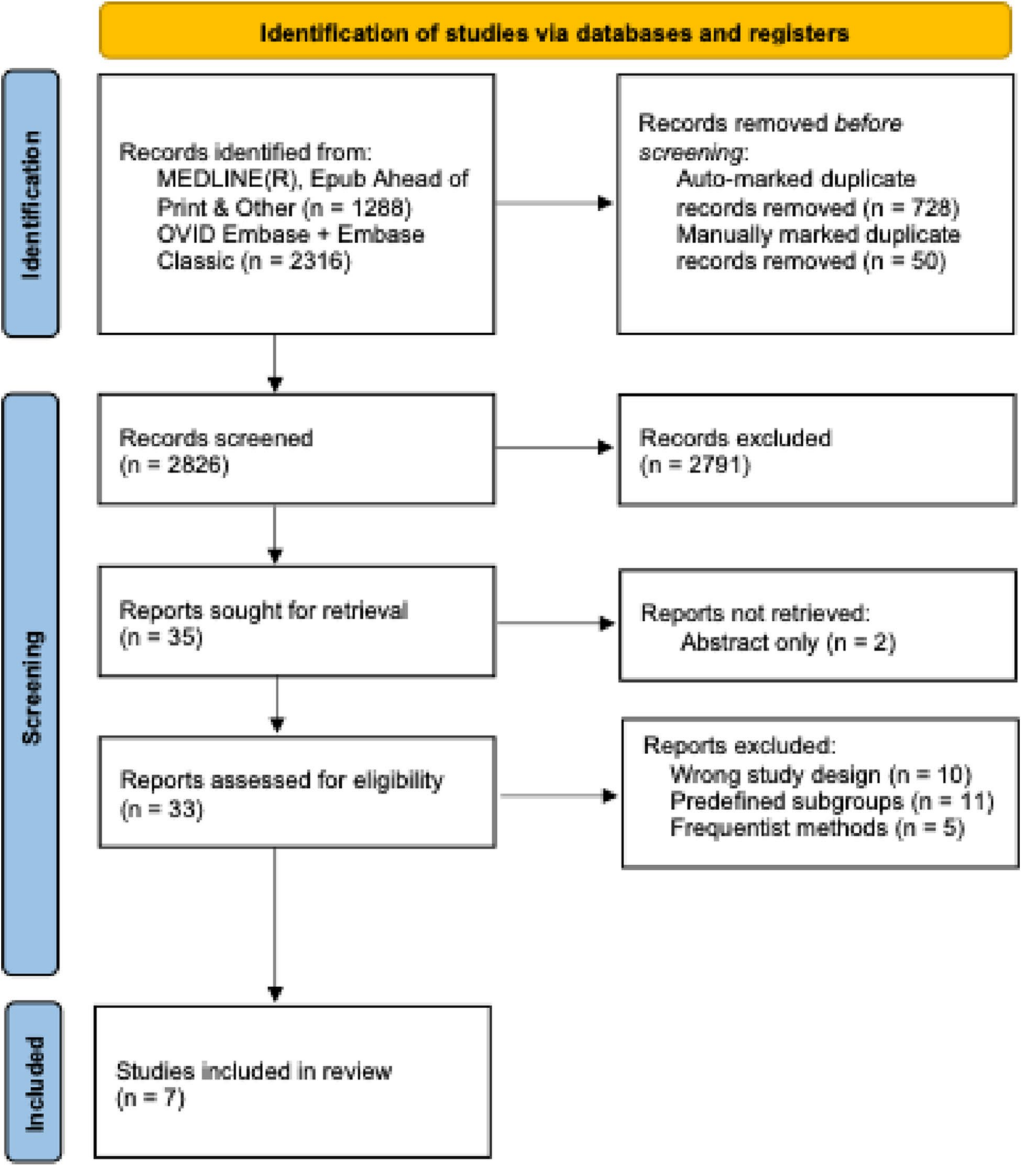
Flow diagram (PRISMA 2020) displaying the systematic review process. Selection phases include identification, screening, and final inclusion, using the inclusion and exclusion criteria in Table 1. *From:* Page MJ, McKenzie JE, Bossuyt PM, Boutron I, Hoffmann TC, Mulrow CD, et al. The PRISMA 2020 statement: an updated guideline for reporting systematic reviews. BMJ 2021;372:n71. 10.1136/bmj.n71

This review was conducted and reported following the PRISMA 2020 guidelines [39]. A PRISMA flow diagram summarizing the study selection is presented in Fig. 1, and a completed PRISMA checklist is provided in the supplementary materials [Additional Table 3]. As all included studies were simulation-based methodological, traditional risk-of-bias assessment tools were not applicable. Instead, we summarized methodological limitations and challenges reported in each study to inform interpretation.

### Data extraction and synthesis

Data extraction was performed using standardized forms developed for this review. One reviewer (RG) extracted the data, and a second reviewer (CG) verified its accuracy. Extracted data included key study characteristics such as outcome type, adaptation strategies, methods for determining HTE subgroups, decision criteria for trial adaptation, and trial phases (I, II, III). We also assessed whether studies addressed diversity and inclusion in subgroup identification—defined here as the consideration of patient characteristics relevant to social determinants of health, including but not limited to race, ethnicity, sex, gender, socioeconomic status, and disability status [40]. This was operationalized by recording whether the study explicitly targeted, stratified by, or discussed these characteristics when identifying or analyzing treatment subgroups. Methodological challenges and limitations reported in the studies were also documented to provide context. Findings were synthesized in a descriptive narrative, with a structured table summarizing study characteristics to identify trends in Bayesian methods for HTE detection within adaptive designs and highlight existing gaps in the literature.

## Results

### Identification of relevant studies

From an initial 3604 records, 2826 unique titles and abstracts were screened (Fig. 1). Of these, 2791 were excluded for not meeting inclusion criteria and two were excluded due to lack of full-text availability. Full-text review was conducted for 33 articles, of which 26 were excluded: 10 did not include adaptive methodology, 11 focused exclusively on predefined subgroups, and 5 relied solely on frequentist statistical approaches. Ultimately, 7 studies met the inclusion criteria for this review [41–47].

### Study characteristics

Across the included studies, five evaluated binary treatment assignments [42–46], while two compared multiple treatment arms [41, 47]. Most classified previously unknown HTE subgroups using molecular biomarkers [41, 43–46], with one study incorporating demographic and geographic variables [42]. The number of covariates considered ranged from 1 to 10. Outcome types varied widely, encompassing binary [41, 44, 45, 47], ordinal [42], continuous [43, 46], time-to-event [45], and joint binary–continuous [47] hierarchical outcomes. Adaptive features included adaptive randomization [41, 42, 44], adaptive enrichment [43, 45, 46], and platform designs [47]. One trial employed a phase I/II framework with a hierarchical outcome [47], while the remaining studies were conducted in phase II settings [41–46]. Aside from one study incorporating geographic variation [40], none examined equity-related variables such as race, gender, socioeconomic status, or disability. A detailed summary of study characteristics is provided in Table 2.

**Table 2:** Summary of study characteristics, arranged by date

*Papers*	*Outcome*	*Adaptation*	*Subgroup determination*	*Decision criteria*	*Bayesian statistical methods*	*Limitations*
***Eickhoff*** **et al*.*** [41]	Binary	Adaptive randomization	Partial least squares logistic regression to classify subjects into biomarker profile groups	Probability for favorable treatment response exceeds pre-specified stopping criterion: biomarker profile group is declared as superior over all other treatment-biomarker group combinations	Bayesian logistic regression model for predicting treatment response; regression coefficients assigned multivariate Normal priors	Design lacks adequate control for type I error, necessitating validation through independent phase III studies to confirm results
***Guo and Zhang*** [42]	Ordinal	Adaptive randomization	Spatially structured random effects with a conditional autoregressive (CAR) prior distribution to identify unknown region-level exposure variables	True utility increases by a certain percentage (minimum clinically meaningful increment): experimental treatment is considered promising compared to standard treatment	Bayesian cumulative probit model for relating efficacy of patient-specific covariates and regional spatial effects to treatment response; region-specific random effects assigned and intrinsic CAR prior, regression coefficients given Normal priors, vague inverse-gamma prior on spatial hyperparameter, and uniform priors for category boundaries	Including too many covariates in the model leads to unstable parameter estimates; dimension reduction needed with multiple biomarkers
***Xu *et al*.*** [43]	Continuous	Adaptive enrichment	Recursive partitioning to divide the covariate space, subgroup selected based on posterior probability of treatment effect being larger than a pre-defined threshold	Predicted probability of improved treatment effect is greater than a pre-defined threshold for the total population and/or a specific subgroup	Bayesian subgroup-identification: Bayesian Random Partition model (BayRP); subgroup-specific treatment effects assigned Beta priors for binary outcomes, or Normal for continuous outcomes; subgroup partitions modeled with a random partition prior	Additional algorithms needed to reduce covariate dimension and adapt methodology for other outcome types, which may be computationally challenging
***Xia *et al*.*** [44]	Binary	Adaptive randomization	Random forest classifier function is used to categorize patients as sensitive or non-sensitive based on biomarker profile	Probability of positive response in the treatment group is larger than a cut-off parameter whose value is tuned to control Type I error rates	Bayesian random forest: aggregate predictions from an ensemble of decision trees, each tree viewed as a sample from a posterior distribution over tree structures; regression coefficients assigned Normal priors, and treatment arm response probabilities given non-informative Beta(1,1) priors at final analysis	Design results in operational complexities that may extend trial duration and complicate interpretation of treatment effect estimates
***Park *et al*.*** [45]	Binary and time-to-event	Adaptive enrichment	Regression model variable selection based on differences in treatment response probabilities/hazard ratio	Probability that a patient will benefit more from treatment (personalized benefit index) is larger than a minimally clinically significant improvement in either response probability or survival	Bayesian regression model for variable selection and Bayesian group sequential test procedure used to derive stopping rules for superiority or futility of treatment; spike-and-slab priors on regression coefficients with latent inclusion indicators following bivariate Bernoulli; priors in intercepts, outcome-specific parameters and subgroup parameters are normal or gamma	Adaptive enrichment design is limited in its scalability for high-dimensional covariates; may restrict the applicability of findings to broader patient populations
***Mu *et al*.*** [47]	Joint binary-continuous	Platform design	Latent subgroup membership inferred using a shared latent class structure linking efficacy and toxicity outcomes	Posterior probability that a treatment is optimal within a latent subgroup exceeds a pre-specified threshold for dose escalation, de-escalation, or arm termination	Hierarchical Bayesian latent class model jointly modeling efficacy (logistic) and toxicity (Emax) outcomes; Dirichlet priors for class proportions; Normal-Inverse Gamma priors for variance parameters; Normal priors for regression parameters with empirically elicited means	Assumes rapid availability of toxicity outcomes; extending model to multiple sensitivity groups would necessitate model selection criteria such as the deviance information criterion
***Maleyeff *et al*.*** [46]	Continuous	Adaptive enrichment	Identification of predictive variables and subgroup thresholds using Bayesian model averaging (BMA) combined with free-knot spline models	Posterior probability that a candidate predictive variable and threshold define a subgroup with a superior mean treatment effect exceeds a pre-specified decision criterion	Bayesian model averaging and spline regression; competing models denote alternative combinations of predictive variables and spline knot placements; posterior model probabilities updated at interim analyses to guide enrichment; right-truncated Poisson priors on number of included biomarker terms and spline knots, uniform priors on variable and knot inclusion indicators, Normal priors on regression coefficients**,** and Inverse-Gamma prior on residual variance	Performance depends on prior specification and number of spline knots; difficulties estimating empirical densities in large biomarker spaces without monotonicity constraints

### Overview of Bayesian methodologies

The Bayesian methodologies proposed across studies varied in complexity and application focus. Xu et al. [43] developed the Bayesian Random Partition (BayRP) model to identify hidden subgroups within complex covariate spaces where treatment effects varied. Using recursive partitioning and posterior probability thresholds, this approach dynamically refined eligibility criteria based on interim data, aligning with precision medicine objectives. Eickhoff et al. [41] combined Bayesian logistic regression with partial least squares logistic regression (PLSLR) for dimensionality reduction, improving biomarker-based subgroup identification and implementing a posterior probability-based stopping criterion for early termination once predefined efficacy thresholds were met. Guo and Zhang [42] extended this framework to account for geographic variability by developing a Bayesian cumulative probit model with spatially structured random effects and conditional autoregressive (CAR) priors, capturing regional correlations in treatment response. Like Eickhoff et al. [41], they used posterior estimates to guide patient recommendations, enhancing detection of hidden HTE driven by unmeasured spatial factors.

Later studies expanded on these foundations by relaxing prior modeling assumptions and accommodating more complex covariate structures. Xia et al. [44] proposed a signature enrichment design with Bayesian response-adaptive randomization (SEDAR), integrating a random forest classifier with Bayesian decision rules. This approach allowed real-time adjustments to treatment allocation probabilities based on accumulating biomarker data, thereby prioritizing subgroups most likely to benefit and improving trial efficiency and power. Park et al. [45] developed a group sequential enrichment design that combined Bayesian regression-based variable selection with group sequential monitoring rules. This design permitted trial enrichment and early stopping based on accumulating hazard ratios and posterior response probabilities, refining trial focus toward biomarker-sensitive subpopulations. Building on this framework, Maleyeff et al. [46] proposed a Bayesian adaptive enrichment design that relaxed Park et al.’s [45] monotonicity and linearity constraints, using Bayesian model averaging with free-knot splines to capture nonlinear treatment–biomarker interactions and define “effective subspaces” where the posterior probability of treatment benefit exceeded a clinically meaningful.

threshold. Similarly, Mu et al. [47] introduced a Bayesian latent‐subgroup platform design that inferred treatment‐sensitive versus insensitive subgroups through a hierarchical model. Subgroup membership was updated iteratively from accumulating efficacy and toxicity data, allowing information borrowing among arms with similar dose–response patterns and improving detection of heterogeneous treatment effects across indications and combination partners.

Across studies, priors were generally specified as non-informative or weakly informative, such as Normal distributions with large variances for regression coefficients [41, 42, 44–47] or Beta(1,1) (i.e., uniform) priors for probabilities [42–44] (Table 2). Study-specific details of each Bayesian HTE detection method are summarized in the supplementary materials [see Additional Table 4].

### Evaluation of operating characteristics and design performance

All seven studies evaluated the performance of their proposed designs via simulation, with performance metrics reflecting both diverse study aims and shared emphases on power, efficiency, and subgroup identification accuracy. Overall, Bayesian methods generally outperformed alternative designs, offering improvements across these areas (Table 3).

**Table 3:** Summary of operating characteristics and/or performance metrics

*Study*	*HTE approach*	*Performance metrics*	*Key findings*	*Comparators*	*Computation and software*
*Eickhoff *et al	Bayesian logistic regression + Partial Least Square Regression (PLSLR)	Sample size efficiency, subgroup identification	Smaller required sample size and optimized efficiency compared to non-Bayesian alternatives	Frequentist logistic regression, non-Bayesian methods for sample size optimization	MCMC^1^ via Block Gibbs sampling with iterative updates; software not specified; no code provided
*Guo and Zhang*	Spatial effects within Bayesian cumulative probit model	Percent of correct selection (PCS), geographic variability	Spatial effects improved treatment accuracy; robust to prior distribution variations	Non-spatial cumulative probit models, generalized linear models	MCMC^1^ via Gibbs sampling in R; code available upon request
*Xu *et al	Bayesian Random Partition (BayRP)	True positive/negative rates, adaptability	Superior subgroup detection and population enrichment for Alzheimer’s trials	K-means clustering, other partitioning methods without Bayesian framework	MCMC^1^ via two-step Metropolis–Hastings sampling; software not specified; no code provided
*Xia *et al	Bayesian Random Forest classifier + Bayesian decision rules (SEDAR)	Power, real-time randomization	Higher power in 12 of 15 scenarios; robust for real-time adjustments	Non-Bayesian random forest models, fixed randomization methods	Bayesian computation details not specified; software not specified; no code provided
*Park *et al	Bayesian regression	Generalized power, subgroup identification	Improved identification of sensitive subgroups for BRCA-mutant ovarian cancer	Frequentist regression models, non-Bayesian subgroup analyses	MCMC^1^ via Stochastic Search Variable Selection (SSVS) with Metropolis–Hastings sampling in R; code provided
*Mu *et al	Bayesian latent subgroup platform design	Correct optimal biological dose (OBD) selection, subgroup identification, patient allocation	Improved identification of sensitive subgroups; higher correct OBD selection rates	Modified co-optimization platform design (COOP) and independent COOP (non-hierarchical) designs	MCMC^1^ via Gibbs sampling in R; code provided
*Maleyeff *et al	Bayesian model averaging with free-knot splines	Power, generalized power, subgroup identification, enrichment accuracy, sample size efficiency	Higher power when nonlinear and nonmonotone effects are present; slightly improved accuracy and subgroup identification	Park et al.’s Bayesian regression approach and a Bayesian adaptive enrichment design with pre-defined predictive biomarkers	Reversible jump (rj) MCMC^1^ via combined Gibbs and Metropolis–Hastings sampler in R; code provided

^1^Markov Chain Monte Carlo (MCMC) is a class of algorithms used to sample from complex probability distributions. These methods rely on constructing a Markov chain whose stationary distribution matches the desired distribution. By iterating the chain and sampling from it, MCMC allows for the estimation of posterior distributions, often used in Bayesian analysis to draw inferences about model parameters

Eickhoff et al. [41], Guo and Zhang [42], and Xia et al. [44] each evaluated adaptive randomization frameworks, but with distinct performance priorities. Eickhoff et al. [41] focused on improving sample size efficiency in subgroup identification, showing that their Bayesian design with PLSLR required fewer participants and enabled earlier stopping compared to a marker-by-treatment Bayesian adaptive randomization design. Guo and Zhang [42] emphasized treatment recommendation accuracy, incorporating spatial structure into a Bayesian cumulative probit model to account for geographic variability and demonstrating robustness to prior assumptions. In contrast, Xia et al. [44] prioritized statistical power, showing that their SEDAR design achieved higher power than standard adaptive designs in 12 out of 15 simulated scenarios, supported by case modelling from the Iressa Pan-Asia Study (IPASS) [48] trial.

Studies applying adaptive enrichment strategies also differed in their evaluation of operating characteristics. Xu et al. [43] emphasized subgroup recovery accuracy, reporting that their BayRP model achieved higher true positive and true negative rates than both Bayesian linear regression and a frequentist tree-based method (GUIDE [49]), highlighting the advantages of Bayesian partitioning in detecting complex treatment-covariate interactions. Building on this, Park et al. [44, 45, 48] incorporated subgroup identification accuracy into a formal inferential framework using generalized power (GP), which jointly reflects the probability of correctly identifying the sensitive subgroup and rejecting the null hypothesis of no treatment benefit. Their Bayesian group sequential enrichment design achieved higher GP for identifying sensitive subpopulations compared with alternative enrichment designs, such as GSED [50], InterAdapt [51], Simon [31], and CGS [31]. Maleyeff et al. [46], extending Park’s framework, demonstrated that their spline-based adaptive enrichment design further improved sensitivity and GP for detecting nonlinear treatment-biomarker relationships while maintaining efficiency. Finally, Mu et al. [47] extended Bayesian adaptivity to a platform setting, showing that their latent-subgroup design accurately distinguished sensitive versus insensitive arms and achieved higher rates of correct optimal biological dose (OBD) selection and patient allocation efficiency compared with analogous non-Bayesian platform designs (mCOOP, iCOOP).

### Distinguishing the role of Bayesian methods in detecting HTE

Although Bayesian methods frequently improved HTE detection and trial efficiency, only one study directly isolated the contribution of Bayesian modeling from the general advantages of adaptive designs. Xu et al. [43] provided a clear Bayesian vs. non-Bayesian comparison, showing their Bayesian partitioning method outperformed a frequentist tree-based method in subgroup recovery. Other studies, including more recent work (Maleyeff et al. [46]; Mu et al. [47]) compared multiple Bayesian adaptive designs rather than Bayesian versus non-Bayesian methods, demonstrating that specific modeling choices such as spline-based enrichment or hierarchical borrowing can further enhance sensitivity, generalized power, and allocation efficiency within Bayesian frameworks. Remaining studies focused on alternative adaptive designs, variants within Bayesian models, or overall trial operating characteristics, making it difficult to disentangle improvements due to Bayesian modeling from those arising from adaptive design features.

### Attention to diversity and inclusion in patient subgroup identification

Across the included studies, demographic or equity-related variables were rarely incorporated into subgroup identification. Only one study, Guo and Zhang [42], explicitly incorporated a demographic variable—geographic location—capturing regional variation in treatment response that may reflect social or environmental determinants of health. All others identified subgroups primarily through continuous or binary biomarkers and did not frame their methods as equity-oriented or prioritize variables such as race, ethnicity, gender, socioeconomic status, disability, or other key social determinants of health.

While.

all studies emphasized the potential of Bayesian approaches for personalized medicine—improving precision in treatment targeting and optimizing trial resources—none connected these strategies to diversity, equity, or ethical considerations, such as fair access to effective treatments across diverse patient populations. Several designs demonstrated methodological flexibility that could support equity-focused analyses, even if not their stated aim: Park et al. [45] used binary biomarkers to define subpopulations for adaptive enrichment, which could extend to social or demographic variables; Xu et al. [43] developed a flexible partitioning model accommodating binary, continuous, ordinal, and categorical covariates, which could feasibly include equity-relevant indicators, and Maleyeff et al. [46] combined spline-based modeling with Bayesian model averaging to capture nonlinear and potentially intersectional subgroup patterns. Despite this adaptability, none operationalized their models for equity, highlighting a clear gap between methodological innovation and its application to advancing equity and inclusion in trial design.

### Limitations and future work reported in reviewed studies

Several methodological limitations were noted across studies by their authors. All relied exclusively on simulation-based evaluations without validation using real patient data, limiting insight into real-world trial implementation [41]. Small to moderate simulated sample sizes contributed to unstable parameter estimates in high-dimensional settings [42], and high-dimensionality also posed challenges for model stability [46] and generalizability [45], especially in adaptive enrichment designs [45]. Although preliminary biomarker screening was used in some designs to mitigate this, such simplifications may reduce applicability to heterogenous populations [43, 45]. The trade-off between modeling flexibility and interpretability was also highlighted, as including many continuous biomarkers without monotonicity constraints can improve sensitivity but limit clinical interpretability [46]. Availability of timely outcomes was another noted limitation, as some designs assumed rapidly observable outcomes to guide adaptations; delayed outcomes would require Bayesian data augmentation and model selection criteria for multiple sensitivity groups [47]. Across studies, the computational intensity of Bayesian models, especially those modelling continuous biomarkers [46], was highlighted as a key barrier to scalability [46], particularly in resource-constrained trial settings [44]. Reproducibility improved over time with R code provided by the three most recent studies [45–47], while earlier studies only offered code upon request [42] or did not specify the software used [41, 43, 44, 47]. Authors called for future research to validate Bayesian HTE detection methods using real patient data [41, 46], to develop computationally efficient, interpretable, and reproducible algorithms [44–46].

## Discussion

This systematic review synthesizes recent methodological advances in Bayesian statistical methods for detecting hidden heterogeneity of treatment effect (HTE) within adaptive clinical trials. Across seven included studies [41–47], Bayesian models were applied to identify patient subgroups with differential treatment response using real-time adaptation strategies, such as adaptive randomization or enrichment. Collectively, the evidence suggests that Bayesian methods can enhance power and precision in detecting HTE, offering a promising but still preliminary framework for more personalized—and potentially more equitable—trial designs. However, the limited number of studies and reliance on simulations constrain generalizability, and direct comparisons with frequentists approaches remain sparse.

### Advantages of Bayesian methods in HTE detection

Building on prior work [36, 52], our findings highlight how Bayesian approaches for subgroup detection in adaptive clinical trials provide greater flexibility, integrate prior knowledge, perform better in small subgroups, and enable more comprehensive modeling of patient characteristics—key advantages for promoting equity.

#### Flexibility in adaptive designs

Bayesian methods enable continuous model updating as data accrue, facilitating real-time modifications to treatment allocation and subgroup classification. This aligns naturally with adaptive trial structures and avoids many statistical complications from repeated interim analyses. Bayesian HTE models can also be embedded within stopping rules for superiority or futility by specifying posterior probability thresholds for overall and subgroup effects. Unlike most adaptive and platform trials, which apply stopping rules to pre-specified strata, Bayesian HTE approaches allow subgroup definitions to emerge dynamically—a concept analogous to basket trials, except that the “baskets” are identified adaptively rather than fixed in advance. Recent work has begun to explore these possibilities [53], marking an emerging area of development. For underrepresented populations, such adaptability ensures that subgroup evidence informs ongoing trial decisions, offering both ethical and practical benefits.

#### Integration of prior knowledge

A defining strength of Bayesian methods is the ability to incorporate of prior information—from expert opinion, literature, or pilot data—into model estimation. In the context of equity, this allows researchers to account for established disparities or underrepresentation when modeling subgroup effects; for example, priors can reflect known differences in treatment response across demographic or social groups. This is especially valuable when data for certain subgroups are sparse and frequentist estimates unstable.

#### Improved power in small samples

Traditional subgroup analyses often suffer from low power, particularly when sample sizes are modest or subgroups are defined post hoc. Bayesian methods can mitigate this by “borrowing strength” across covariates or trial arms through hierarchical or partial pooling approaches [10, 36, 47]. This framework, used by Mu et al. [47], to share information among arms with similar dose–response patterns, improves the precision of subgroup effect estimates. As a result, Bayesian designs particularly valuable for early-phase trials, rare diseases, and equity-focused research with limited recruitment.

#### Simultaneous subgroup modeling

Unlike many frequentist approaches that test covariates sequentially or in isolation, Bayesian models allow simultaneous estimation of multiple covariates and their interactions, providing a multidimensional view of treatment heterogeneity that reflects the interplay of clinical, biological, and social factors. Maleyeff et al. [46], illustrated this flexibility using Bayesian model averaging with free-knot splines; Xu et al.’s [43] developed a partitioning model that accepts categorical, ordinal, and continuous covariates; and Park et al. [45] used binary markers that could represent non-biological subgroup definitions. However, none of the included studies incorporated equity-related variables such as race, gender, socioeconomic status, or disability–likely because equity was not their stated focus. This omission highlights a key opportunity: while the reviewed methods focus on clinical or biomarker variables, they can readily accommodate equity-relevant factors [43].Explicit integration of equity variables could extend Bayesian HTE frameworks to promote inclusion rather than inadvertently reinforce disparities.

### Challenges for broader application of Bayesian HTE detection methods

Despite these strengths, several challenges limit the adoption of Bayesian HTE methods in real-world trials. M

odel performance depends heavily on prior specification, which can influence results—particularly in small or noisy datasets. While informative priors can improve power by shrinking estimates toward plausible values, they risk bias if not well justified; conversely, weak or noninformative priors provide flexibility but may yield unstable subgroup detection [54].

This issue is especially relevant when equity is a goal: without deliberate inclusion of social or demographic priors, models may overlook disparities or reinforce status quo assumptions. Only two studies conducted sensitivity analyses to assess robustness to assumptions on prior assumptions [42, 47]. Similarly, covariate selection—often guided by expert judgment—can embed implicit bias if equity-related factors are not considered. Prior elicitation techniques offer one solution [55], allowing clinical or community expertise to formally shape model structure, yet only one reviewed study applied elicitation procedures [41, 42, 47].

Evaluations of Bayesian HTE detection methods are further constrained by their reliance on simulation studies and inconsistent performance metrics. While simulations allow controlled testing of methodological properties, they cannot fully capture real-world complexities such as data heterogeneity, missingness, or unmeasured confounding [54]—factors especially salient when modeling outcomes in underserved populations. Performance metrics also varied widely: [41]some emphasized sample size efficiency [41, 42] others adaptive accuracy [42], and others generalized power [45, 46]. This variability hampers comparability and standardization, limiting consensus on what constitutes a “successful” design.

In addition, high-dimensional Bayesian models that adapt in real-time require advanced statistical expertise, custom software, and substantial computing resources, creating barriers for many research teams. Code was unavailable for over half of the reviewed studies, compounding reproducibility issues.

Finally, the small number of studies reflects a field still in early development. While these models can uncover latent subgroups, their equity impact depends on representation in the underlying data; Bayesian flexibility cannot compensate for incomplete or biased datasets. Without intentional inclusion of equity considerations in both design and data collection, even sophisticated methods risk perpetuating disparities rather than addressing them.

### Limitations of the current review

This review provides a focused synthesis of recent methodological advances but has several limitations. It was conducted as a rapid synthesis and not pre-registered, which may limit transparency. The included studies–restricted to adaptive trial designs– represent a narrow scope and may not reflect all emerging Bayesian HTE methods. Although all studies proposed novel frameworks, the lack of available code precludes a formal comparison of the methods outlined across papers. Future work should conduct quantitative evaluations of these designs to more robustly assess performance and identify best practices for real-world application.

### Practical considerations for researchers

To advance the use of Bayesian HTE detection in adaptive trials, especially from an equity perspective, future work should:

1. **Develop Standardized Guidelines**: Establish clear protocols for prior selection, covariate justification, and sensitivity analyses—ideally with an equity lens—to improve transparency and reduce bias. Guidelines should also define consistent operating characteristics (e.g., generalized power) to standardize evaluation and enable comparability across studies, building on existing frameworks such as the CONSORT and SPIRIT extensions for adaptive trials.
2. **Optimize Trial Designs**: Ensure methods are computationally feasible and robust to small samples, akin to early-phase or resource-constrained settings. Simulation tools such a *Stan, JAGS* or *NIMBLE* can facilitate design optimization.
3. **Expand Applications**: Apply Bayesian HTE methods to basket or SMART trials that evaluate diverse populations and interventions.
4. **Prioritize Real-World Validation**: Apply models to patient data from ongoing adaptive trials (e.g., REMAP-CAP, I-SPY) to assess generalizability, scalability and utility.
5. **Enhance Collaboration**: Strengthen partnerships among statisticians, clinicians, and regulators to integrate Bayesian methods within existing adaptive trial frameworks and regulatory pathways (e.g. FDA, EMA).
6. **Promote Transparency and Access**: Develop open-source software and shared code repositories from published studies, provide user-friendly interfaces, and offer accessible documentation to lower barriers to adoption, particularly for non-specialists conducting community-based or equity-focused trials.

To guide trial designers, Table 4 summarizes the practical implications of Bayesian HTE methods across common adaptive trial features, outlining recommended approaches, advantages, limitations, and how each feature might support—or miss—opportunities to advance health equity. This synthesis helps align statistical innovations with inclusive design goals and real-world implementation.

**Table 4:** Practical implications of Bayesian HTE detection methods for trial design and equity

*Trial feature*	*Recommended Bayesian method*	*Key advantages*	*Challenges*	*Equity considerations*
*Small sample size*	Hierarchical models, informative priors	Improved power, parameter sharing	Prior specification sensitivity	May improve subgroup detection in rare diseases or underserved populations
*Multiple biomarkers*	PLSLR, Bayesian variable selection, Bayesian model averaging	Dimensionality reduction	Interpretability, convergence, computational resources	Equity depends on inclusive biomarker selection and validation across groups
*Geographic variation*	Spatial CAR priors	Adjusts for unmeasured confounding	Requires spatial data	Supports detection of place-based inequities and regional access disparities
*Evolving subgroups*	Random partition models	Flexibility, real-time updating	High computational cost	Allows identification of emergent patterns in marginalized subgroups
*Allocation or dose decisions*	Adaptive randomization, latent-subgroup platform	Power, personalization, efficacy	Trial duration extension, rapid outcomes needed	Can prioritize treatment for underserved or high-risk populations

## Conclusion

Bayesian methods offer promise for detecting hidden treatment heterogeneity in adaptive clinical trials through dynamic modeling, improved precision, and responsiveness to emerging evidence. While well suited to personalized and inclusive care, current evidence is limited and largely simulation-based. Real-world validation comparative evaluation, and intentional design choices that prioritizing diversity are needed. Greater standardization, validation, and interdisciplinary collaboration will be essential to translating Bayesian HTE detection into equitable, next-generation trial design.

## Supplementary Information

Additional file 1. Full search strategy.

Additional file 2. PRISMA 2020 checklist.

Additional file 3. Detailed explanation of HTE modelling strategies.

## Data Availability

This study is a systematic review and does not involve the collection of primary data. All data used in this review were obtained from publicly available sources, published literature, or databases as cited in the manuscript. Any restrictions on data availability are due to third-party copyright or database access limitations.
