# Supplementary material for: Who benefits? Uncovering hidden heterogeneity of treatment effects in adaptive trials using Bayesian methods: a systematic review
Source: Trials. 2025 Nov 25;26:593. doi: 10.1186/s13063-025-09291-x (PMC12751210; doi:10.1186/s13063-025-09291-x)
Supplement: Supplementary file 1 — Additional file 1. Full search strategy. [file 13063_2025_9291_MOESM1_ESM.docx]

**Additionaly Table 1. Complete search strategy terms**

1. Randomized control trial or pragmatic clinical trial

((Randomized Controlled Trial or Pragmatic Clinical Trial).pt. or exp "Randomized Controlled Trials as Topic"/ or Controlled clinical trial.pt. or exp controlled clinical trials as topic/ or clinical trial.pt. or exp clinical trials as topic/))

OR (("Randomi?ed Controlled Trial?" or "Controlled Clinical Trial?" or "Randomi?ed Clinical Trial?" or "clinical trial" or RCT? or CCT?).ti,ab,kf.)

1. Statistical methodology or design

(Research design/ or (((method* or design?) and (stud* or trial?)) or methodolog* or procedure? or technique? or simulation stud*).ti,ab,kf.)

OR ((instrumentation or methods or design or Bayesian design or simulation study).sh.) OR (Bayes Theorem/ or ((Theorem* or theory* or forecast* or analy* or approach* or method$ or estimat* or predict* or calculat* or statistical model$ or analysis model* or study design* or design) adj4 (Bayes or Bayesian*)).ti,ab,kf.)

OR ("Posterior probability" or "Posterior distribution" or "Prior distribution" or "Uninformative prior" or prior or "Credible interval" or "Hierarchical model*" or "Dynamic borrowing" or "Predictive probability").ti,ab,kf.)

1. Heterogeneity of treatment effect

((subset* or variab* or subgroup? or "sub-group?" or "treatment arm ") and select*).ti,ab,kf.

OR (Treatment Effect Heterogeneity/ or (Heterogene* and ("patient population*" or "sample group?" or "treatment effect*")).ti,ab,kf.)

1. Adaptive clinical trial

(exp "Adaptive Clinical Trials as Topic"/ or "Adaptive Clinical Trial".pt. or (("adaptive signature" or "adaptive enrichment" or adaptive or "adaptive clinical" or prospective* or outcome-adaptive) adj2 (randomi* or trial* or design* or "study design*")).ti,ab,kf.
