# Supplementary material for: Who benefits? Uncovering hidden heterogeneity of treatment effects in adaptive trials using Bayesian methods: a systematic review
Source: Trials. 2025 Nov 25;26:593. doi: 10.1186/s13063-025-09291-x (PMC12751210; doi:10.1186/s13063-025-09291-x)
Supplement: Supplementary file 3 — Additional file 3. Detailed explanation of HTE modelling strategies. [file 13063_2025_9291_MOESM3_ESM.docx]

**Additional Table 3. Detailed explanation of HTE modelling strategies**

| **HTE Modeling Strategy** | **Description of How HTE is Detected** | **Modeling Approach** |
| --- | --- | --- |
| **Bayesian Logistic Regression with PLSLR – Eickhoff et al. (2010)** | This approach uses **Bayesian logistic regression** to model how **biomarkers** influence treatment response, incorporating prior knowledge to refine estimates. **PLSLR** reduces the complexity of multiple biomarkers, identifying the most important ones linked to treatment success. The model then estimates the likelihood of treatment benefit for different subgroups, helping to focus on those most likely to respond. If a subgroup shows strong benefits early on, the trial can stop early to save resources. | **Bayesian logistic regression** models biomarker-treatment relationships with prior knowledge, refining estimates. |
| **Bayesian Cumulative Probit Model with Spatial Effects – Guo & Zhang (2019)** | This method focuses on **geographic variability** in treatment response. It considers regional effects by modeling how nearby locations may have similar responses. **Spatial effects** are captured using **autoregressive priors**, helping to identify location-based subgroups that may respond differently to the treatment due to environmental or regional factors. This approach allows for more accurate detection of HTE in patients from different geographic areas. | **Bayesian regression** models treatment response with **spatial effects** to account for regional treatment variability. |
| **Bayesian Random Partition (BayRP) – Xu et al. (2020)** | This method works by **splitting the patient group** into smaller, more similar subgroups based on key characteristics (like age or health condition). It **keeps splitting** the group until it finds the subgroups where the treatment has the most different effects. The algorithm **tests different groupings** and looks for those where treatment works better or worse for certain types of patients. This way, it can detect which subgroups benefit the most, ensuring the trial focuses on the most promising patients. | **Uses recursive partitioning (CART method)** to split data into subgroups based on characteristics, detecting where treatment effects differ. |
| **Bayesian Adaptive Randomization – Xia et al. (2021)** | This method uses a **random forest classifier**, which is a collection of **decision trees** that work together to identify patterns in patient data (such as biomarkers). Each decision tree creates a separate pathway for grouping patients based on their likelihood of responding to treatment. The random forest combines the results of multiple trees to make more accurate decisions about which patients are most likely to benefit from the treatment. After identifying the most promising subgroups, **Bayesian decision rules** are used to adjust the trial in real time, prioritizing patients who are most likely to benefit. This approach helps focus the trial on the right patients, improving both efficiency and the likelihood of success. | **Random forest classifier (CART method)** detects patterns and prioritizes subgroups most likely to benefit, with **Bayesian decision rules** to adjust trial in real time. |
| **Bayesian Regression with Group Sequential Enrichment – Park et al. (2022)** | This method uses **statistical models** to identify key variables that predict how patients will respond to treatment. It **focuses the trial** on the subgroups that are most likely to benefit. By checking treatment responses regularly, the trial can **stop early** if a subgroup shows particularly good results. The method makes sure resources are used wisely by focusing on the most responsive groups, adjusting the trial as it progresses based on **real-time data**. | **Bayesian regression** identifies predictive variables and adjusts the trial focus in real time to prioritize promising subgroups. |
| Bayesian Latent Class Dose-Finding Design – Mu et al. (2024) | This design identifies hidden (latent) patient subgroups that respond differently to treatment doses, based on both side effects and benefits. By **analyzing these two outcomes together**, it can uncover patterns that wouldn’t be visible otherwise. As more patient data come in, the model updates who belongs to each subgroup and **adjusts dose levels** accordingly, helping to find the best dose for each type of patient. | **Bayesian latent class model** groups patients with similar treatment responses and balances safety and efficacy to guide adaptive dose transitions. |
| Bayesian Model Averaging with Free-Knot Splines – Maleyeff et al. (2024) | This approach first identifies an “effective subspace” — the combination of biomarkers that best defines patients most likely to benefit — by fitting **flexible (spline) models** across different biomarker patterns. It then **averages across these models** to avoid overfitting and checks how common the subgroup is in the trial (if it’s too small, the trial may stop early). Finally, it estimates the treatment effect within that subgroup and decides whether to stop for success or futility. This helps the trial focus on the most promising patients while avoiding unreliable results. | **Bayesian model averaging** compares multiple models to find the best-fitting subgroup patterns to identify predictive biomarkers and optimal subgroup thresholds. |
